# Acute Effects of Nitrogen Dioxide on Cardiovascular Mortality in Beijing: An Exploration of Spatial Heterogeneity and the District-specific Predictors

**DOI:** 10.1038/srep38328

**Published:** 2016-12-02

**Authors:** Kai Luo, Runkui Li, Wenjing Li, Zongshuang Wang, Xinming Ma, Ruiming Zhang, Xin Fang, Zhenglai Wu, Yang Cao, Qun Xu

**Affiliations:** 1 Department of Epidemiology and Biostatistics, Institute of Basic Medical Sciences Chinese Academy of Medical Sciences, School of Basic Medicine Peking Union Medical College, Beijing 100005, China; 2 Centre of Environmental and Health Sciences, Chinese Academy of Medical Sciences, Peking Union Medical College, Beijing 100005, China; 3 College of Resources and Environment, University of Chinese Academy of Sciences, Beijing 100049, China; 4 State Key Laboratory of Resources and Environmental Information System, Institute of Geographic Sciences and Natural Resources Research, Chinese Academy of Science, Beijing 100101, China; 5 Chinese Research Academy of Environmental Sciences, Beijing 100012, China; 6 Unit of Biostatistics, Institute of Environmental Medicine, Karolinska Institutet, Stockholm 17177, Sweden; 7 Clinical Epidemiology and Biostatistics, School of Medical Sciences, Örebro University, Örebro 70185, Sweden

## Abstract

The exploration of spatial variation and predictors of the effects of nitrogen dioxide (NO_2_) on fatal health outcomes is still sparse. In a multilevel case-crossover study in Beijing, China, we used mixed Cox proportional hazard model to examine the citywide effects and conditional logistic regression to evaluate the district-specific effects of NO_2_ on cardiovascular mortality. District-specific predictors that could be related to the spatial pattern of NO_2_ effects were examined by robust regression models. We found that a 10 μg/m^3^ increase in daily mean NO_2_ concentration was associated with a 1.89% [95% confidence interval (CI): 1.33–2.45%], 2.07% (95% CI: 1.23–2.91%) and 1.95% (95% CI: 1.16–2.72%) increase in daily total cardiovascular (lag03), cerebrovascular (lag03) and ischemic heart disease (lag02) mortality, respectively. For spatial variation of NO_2_ effects across 16 districts, significant effects were only observed in 5, 4 and 2 districts for the above three outcomes, respectively. Generally, NO_2_ was likely having greater adverse effects on districts with larger population, higher consumption of coal and more civilian vehicles. Our results suggested independent and spatially varied effects of NO_2_ on total and subcategory cardiovascular mortalities. The identification of districts with higher risk can provide important insights for reducing NO_2_ related health hazards.

Plenty of epidemiological studies have provided substantial evidence that increase in concentration of ambient nitrogen dioxide (NO_2_), a commonly used surrogate of traffic-related air pollutants, was associated with elevated risk in all nature causes^1^, respiratory^2^ and cardiovascular^3^ mortality around the world. Although previous studies have suggested independent effects of NO_2_ even after adjustment for co-pollutants^4^,^5^,^6^, whether these associations reflect true effects of NO_2_ is still a matter of debate^7^.

Recently, King’s College London reported that total mortality burden of long term exposure to NO_2_ was estimated 88,113 life years lost, equivalent to 5,879 premature deaths in London^8^. Notably, the numbers for acute or chronic effects of NO_2_ could be larger in Beijing, China, because of the serious air pollution, higher traffic emission and heavy burden of chronic disease^9^,^10^,^11^. Previous studies in Beijing were mainly focused on the relationships between particulate matters less than 10 micro meters in diameter (PM_10_) and all causes or respiratory mortality^5^,^12^,^13^, however, few investigated the acute effects of NO_2_ on total and subcategory cardiovascular mortalities^14^.

There is a growing body of evidence that shows greater spatial variation in distribution of NO_2_ within city compared with PM_10_^12^,^15^, as well as spatial variation among at-risk individuals across city^16^. Our recent study observed spatial heterogeneity of effects of air pollutants, including NO_2_ and PM_10_, on respiratory mortality in 16 districts in Beijing^5^. Additionally, Zhang *et al*.^12^ and Xu *et al*.^13^ also reported the spatial variation in effects of PM_10_ on cardiovascular mortality. However, seldom did studies investigate the spatial variation in the effects of NO_2_ on total and subcategories cardiovascular mortality. No study has investigated the underlying spatial heterogeneity of NO_2_-related health hazards using district-level indicators in Beijing, which might provide important insights for public health policymakers to identify vulnerable districts and allocate health resources efficiently.

The specific objectives of our study were, firstly, to explore the associations between NO_2_ and total cardiovascular, cerebrovascular and ischemic heart disease mortalities; secondly, to investigate the spatial heterogeneity of the associations across the 16 districts in Beijing; and thirdly, to identify the district-level indicators that might contribute to the spatial pattern of the association.

## Results

### Descriptive results

**Figure 1:**
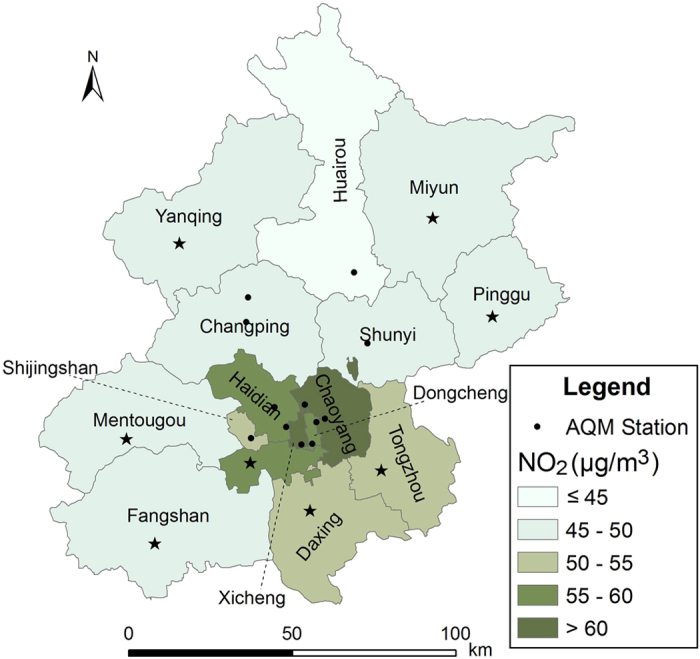
Distribution of the 12 national air quality monitoring (AQM) stations and mean NO_2_ concentrations across the 16 administrative districts in Beijing from 2009–2010. The pentangle asterisks represent the districts with predicted NO_2_ concentration. (The map was created using ArcGIS Desktop version 9.3, the ESRI company, California, USA, URL: http://www.esri.com/).

Table 1 summarizes the cause-specific mortality, air pollution and meteorological conditions in Beijing from 2009 through 2010. The citywide medians of daily concentrations of NO_2_, PM_10_ and CO were 50.41 μg/m^3^, 107 μg/m^3^ and 1.24 mg/m^3^, respectively. Figure 1 shows the distribution of the 12 national air quality monitoring stations in Beijing and the means of daily concentrations of NO_2_ across 16 districts. The first two highest values of NO_2_ level were observed in Xicheng (64.09 μg/m^3^) and Chaoyang (60.42 μg/m^3^) district. In general, the districts in the centre of the city had higher concentration of NO_2_ compared with the suburban districts (Fig. 1). The citywide means of daily deaths of cardiovascular, cerebrovascular, ischemic heart disease were 102.41, 47.53 and 46.15, respectively (Table 1). Supplemental Figure S1 shows the variation of daily mortality rate of total cardiovascular, cerebrovascular and ischemic heart disease among the 16 districts. The highest mortality rates for the three interested outcomes were found in Pinggu (1.25 per 100 thousands), Pinggu (0.82 per 100 thousands) and Mentougou (0.64 per 100 thousands) district, respectively. The lowest rates for the three outcomes were all observed in Haidian district, with values of 0.33 per 100 thousands, 0.12 per 100 thousands and 0.18 per 100 thousands, respectively. The Spearman’s correlation coefficients of air pollutants and meteorological factors are reported in Supplemental Table S2. The coefficients between NO_2_ and meteorological factors were small. Daily average temperature was negatively associated with NO_2_. Distribution of daily concentrations of NO_2_, PM_10_ and CO of 16 districts were displayed in Supplemental Figure S2. Additionally, information on the selected district-specific demographic and socio-economic indicators were summarized in Supplemental Table S1.

### Citywide and district-specific effects of NO_2_ on total and subcategory cardiovascular mortality

**Figure 2:**
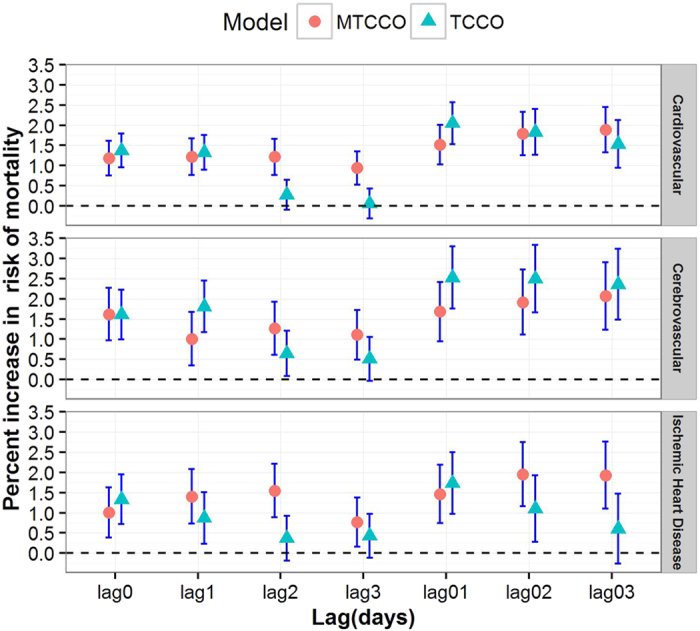
Citywide effect estimates of short term exposure to NO_2_ on cardiovascular, cerebrovascular and ischemic heart disease mortalities and the lag patterns of the associations using multilevel time stratified case-crossover (MTCCO) design and conventional time stratified case-crossover (TCCO) design, respectively. For single lag days, lag0 refers to current day, lag1 refers to previous one day, the rest single lag days were defined in the same way. For multi-lag days, lag01 refers to moving average of current day (lag0) and previous one day (lag1), the rest multi-lag days were defined in the same way.

Figure 2 shows the associations between NO_2_ and total and the two subcategory cardiovascular moralities using single pollutant models with different lag structures based on the multilevel time stratified case-crossover (MTCCO) design and the conventional time stratified case-crossover (TCCO) design. For the results from MTCCO design, the estimated effects of NO_2_ on cardiovascular, cerebrovascular and ischemic heart disease mortalities based on multi-day lags were, in general, higher than those based on the single day lags. We found stronger associations of cause specific mortality for cerebrovascular and ischemic heart diseases, compared with those for total cardiovascular disease. For a 10 μg/m^3^ increase in daily average concentration of NO_2_, the strongest and statistically significant effects (presented as percent increase in mortality rate and corresponding 95% CI) for total cardiovascular, cerebrovascular and ischemic heart disease mortalities were observed at lag03 (1.89%, 95% CI: 1.33–2.45%), lag03 (2.07%, 95% CI: 1.23–2.91%) and lag02 (1.95%, 95% CI: 1.16–2.72%), respectively. In contrast, the lag patterns of the results from TCCO design were inconsistent with those from MTCCO, with the strongest effects of NO_2_ found at lag01 for all the three outcomes. But the pattern of effects from single lag days and multi-day lags was similar to that from MTCCO, namely higher estimates in multi-day lags than that of single lag days. The 95% CIs of the estimates at multi-day lags from MTCCO were narrower than those based on TCCO design, although those CIs were partly overlapped for two designs. Thus, we used the lag structures that with the largest effect estimates of NO_2_ from MTCCO design in the following analyses. As illustrated in Supplemental Figure S3, associations from single-pollutant models based on MTCCO design were generally attenuated in multi-pollutant models. But NO_2_ was still positively and significantly associated with cardiovascular and ischemic heart diseases after adjusting for PM_10_ and CO in both two-pollutant and three-pollutant models, except for cerebrovascular mortality. Additionally, the lag pattern of citywide NO_2_ effects from TCCO and MTCCO after adjusting for temperature for four days (lag03) was similar to our primary results (see Supplemental Figure S4).

**Figure 3:**
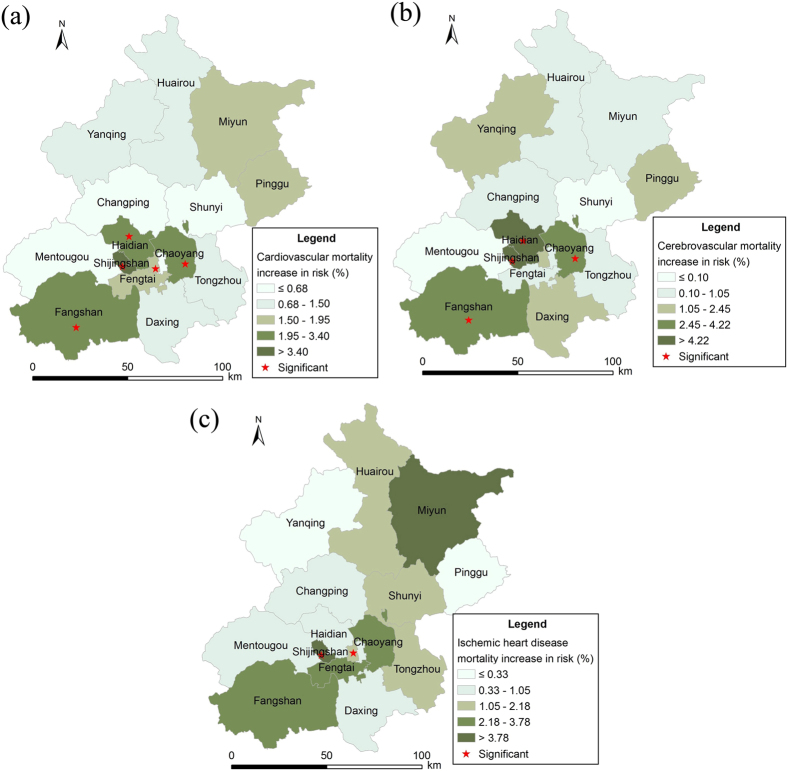
Associations between total cardiovascular (**a**), cerebrovascular (**b**), ischemic heart disease (**c**) mortalities and NO_2_ across the 16 districts in Beijing in terms of percentage increase in daily mortality per 10 μg/m^3^ increase in daily NO_2_ concentration. The exposure metrics of NO_2_ for three outcomes of interest were lag03, lag03 and lag02, respectively. (The map was created using ArcGIS Desktop version 9.3, the ESRI company, California, USA, URL: http://www.esri.com/).

Besides the observed significant estimates of NO_2_ on citywide cardiovascular, cerebrovascular and ischemic heart disease mortality, we also found the spatial variation of the associations across the 16 districts and different spatial patterns for the three outcomes. For total cardiovascular mortality, as depicted in Fig. 3(a), we found largely varied estimates ranged from 0.05% (95% CI: −3.75–4.01%) in Mentougou district to 6.32% (95% CI: 2.18–10.62%) in Shijingshan district. The significant associations were only observed in five districts, including Shijingshan, Fangshan, Chaoyang, Haidian and Xicheng district, with estimates of 6.32% (95% CI: 2.18–10.62%), 3.4% (95% CI: 1.27–5.56%), 3.10% (95% CI: 1.45,4.76%), 2.50% (95% CI: 0.52–4.51%) and 1.8% (95% CI: 0.16–3.46), respectively. For cerebrovascular mortality, the largest effect was observed in Shijingshan district with estimate of 7.13% (95% CI: 0.94–13.69%), meanwhile, the smallest one was 0.10% (95% CI: −2.98–3.29%) observed in Shunyi district, and significant associations were found in four districts [see Fig. 3(b)]. For the ischemic heart disease mortality, we found statistically significant estimates in two districts, including Shijingshan (8.76%, 95% CI: 2.75–15.13%) and Chaoyang (2.80%, 95% CI: 0.51–5.14%) district, while most other districts had non-significant associations [see Fig. 3(c)]. We also observed negative effects in Mentougou district, −0.56% (95% CI: −6.22–5.44%), for cerebrovascular mortality and Pinggu district, −2.54% (95% CI: −8.10–3.36%) for ischemic heart disease, but both were statistically non-significant.

### Associations between district-specific effects and district-level indicators

Of the 28 indicators from six predefined dimensions tested in univariate analyses, only few indicators were statistically significantly associated with spatial pattern of the district-specific associations between NO_2_ and three interest outcomes (see Supplemental Table S3). Specifically, 6 indicators, i.e. gross domestic products (GDP), civilian vehicles, coal consumption, population, population of aged ≥65 years and illiterate population of aged ≥15 years were statistically significantly associated with spatial pattern of NO_2_ related total cardiovascular and cerebrovascular mortalities (Table 2). Additionally, the married percentage was negative associated with risk of NO_2_ related cerebrovascular mortality. In contrast, only illiterate population was associated with spatial pattern for NO_2_ related ischemic heart disease mortality, with estimate of 1.07% (95% CI: 0.02–2.12%). Supplemental Table S4 provides the Pearson’s correlation coefficients of the indicators with significant coefficients in the univariate analyses for the three interest outcomes. In general, there exists strong and statistically significant correlation between these indicators, which indicates the collinearity among the indicators in the multivariable analyses.

**Figure 4:**
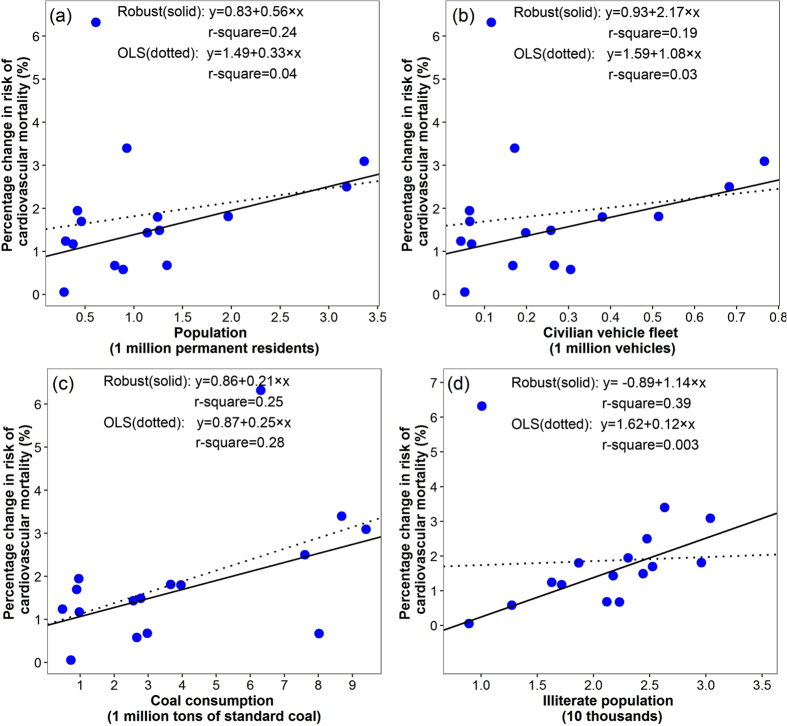
The relationships between district-specific NO_2_-related cardiovascular mortality risk, measured as percentage change in mortality risk per 10 μg/m^3^ increase in four days moving average (lag03) of NO_2_ concentrations and district level indicators in univariate models: (**a**) population, (**b**) civilian vehicles fleet, (**c**) coal consumption, and (**d**) illiterate population. Solid lines were fitted using robust regression models, and dotted lines were fitted using ordinary least squares (OLS) linear regression models.

**Figure 5:**
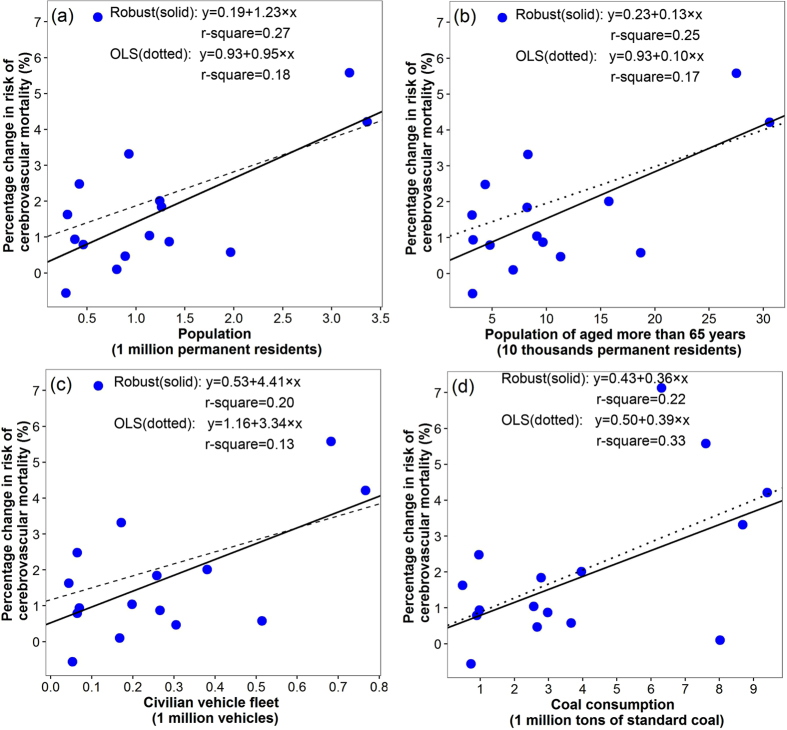
The relationships between district-specific NO_2_-related cerebrovascular mortality risk, measured as percentage increase in mortality risk per 10 μg/m^3^ increase in four days moving average (lag03) of NO_2_ concentrations and district level indicators in univariate models: (**a**) population, (**b**) aged population, (**c**) civilian vehicles fleet, and (**d**) coal consumption. Solid lines were fitted using robust regression models, and dotted lines were fitted using ordinary least squares (OLS) linear regression models.

**Figure 6:**
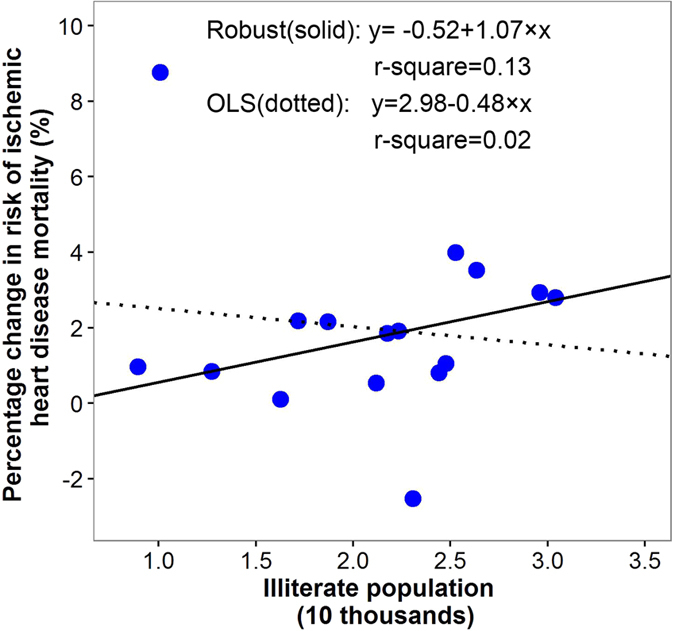
The relationships between district-specific NO_2_-related ischemic heart disease mortality risk, measured as percentage increase in mortality risk per 10 μg/m^3^ increase in three days moving average (lag02) of NO_2_ concentrations, and district-specific illiterate population in univariate model. Solid line was fitted using robust regression model, and dotted line was fitted using ordinary least squares (OLS) linear regression models.

After adjusting for the collinearity using principal component analysis (results were shown in Supplemental Tables S5–10 and Figures S5–6), the multivariable robust regression analyses were conducted for spatial patterns of NO_2_ related cardiovascular and cerebrovascular disease mortality risks. Results showed that all the magnitudes of coefficients were attenuated and GDP and population of aged ≥65 years become non-significant for cardiovascular mortality, meanwhile, the coefficients of GDP, illiterate population and marriage percentage for NO_2_ related cerebrovascular mortality risk become non-significant (Table 2). We did not perform the multivariable analyses for NO_2_ related ischemic heart disease mortality risk, because only one indicator (illiterate population) was statistically significant in the univariate analyses. Besides, the scatter plots (Figs 4, 5, 6) of the relations between the district-specific indicators and NO_2_ related mortality risks in multivariable analyses show that the trend of the lines based on ordinary least squares (OLS) regression were greatly affected by the outliers and extreme values when compared with lines based on the robust regression, which were more obvious in Fig. 4(d) and Fig. 6. Meanwhile, the R-squares from robust regression were generally higher than those of OLS (Figs 4, 5, 6). Thus, the results based on the robust regression were more stable than those from OLS.

## Discussion

In present study, we found that short term exposure to NO_2_ was associated with elevated risk of total cardiovascular, cerebrovascular and ischemic heart disease mortalities even after adjusting for co-pollutants. Higher estimates were found for the latter two outcomes. The results of our study provide evidence of spatially heterogeneous effects of NO_2_ across the 16 administrative districts in Beijing, and several district-specific indicators were associated with the spatial patterns of the risk.

### Estimates of MTCCO and TCCO design

Air pollution data from single monitoring station or average value from several monitoring stations were commonly used as the exposure metric in studies on citywide health effects of air pollution. However, this metric cannot represent the citywide exposure accurately, especially for mega cities where spatial variation of air pollution, such as NO_2_, exists across the geographical area, and might introduce measurement error into the effect estimation and understatement the uncertainty in effect. In our study, we modelled the citywide effects with multilevel time stratified case-crossover design based on exposure metrics at district-level. We found that the 95% CIs of the estimated citywide effects of NO_2_ in multilevel design were narrower than those in the conventional time stratified case-crossover design, although the point estimates from the former were slightly smaller than those from the latter at some multi-day lags. Similar patterns were also observed in other studies^12^,^13^, for example, Zhang *et al*.^12^ reported higher estimates of PM_10_ effects and narrower 95% CIs using generalized additive mixed model (GAMM), which was based on exposure metrics at district-level, compared with those from generalized additive model (GAM). However, the observed narrower 95% CIs might be due to not only the spatial variation of NO_2_ but also the clustering of air pollutant related health outcomes. Prior studies have shown that failure to take into account the clustering of health outcomes of air pollutants would lead to understatement of uncertainty in estimates and may have great impacts on the weak association between air pollutants and mortality^17^. Although sufficient evidence of clustering in NO_2_ related mortality lacked in our study, the application of multilevel time stratified case-crossover design could account for at least part of the uncertainty in the estimation of NO_2_ effects. Therefore, the results from the multilevel design or others that take into account the spatial variation of air pollution were superior to those based on the citywide exposure metrics when conducting citywide air pollution study in mega cities^18^, such as Beijing.

### Acute effects of NO_2_ on cardiovascular mortalities

In general, the findings of citywide associations between NO_2_ and total and subcategories cardiovascular mortalities in present analyses were consistent with those reported by previous investigations^19^,^20^. For cardiovascular mortality, our analysis indicated significant increase (1.89% at lag03; 95% CI: 1.33–2.45%) in total cardiovascular mortality, which was similar to those of other studies conducted in China including single-city studies in Guangzhou^21^ (1.81% at lag01; 95% CI: 1.20–2.41%), Wuhan^22^ (2.12% at lag01; 95% CI: 1.18–3.06%), multicity studies in Pearl River Delta of south of China^23^ (2.12% at lag1–2; 95% CI: 1.58–2.65%) and the CAPES in 17 Chinese cities^24^ (1.80% at lag01; 95% CI: 1.00–2.59%). However, our estimates were greater than those from the studies conducted in Shanghai^15^ (1.01% at lag01; 95% CI: 0.55–1.47%) and Hong Kong^19^ (1.23% at lag01; 95% CI: 0.64–1.82%). The inconsistency and difference between ours and aforementioned results might be partly explained by the difference in study period, population susceptibility, methodology and exposure measurement. Specifically, our citywide estimate of NO_2_ related cardiovascular mortality risk was based on the district-specific concentration, while the estimates from Shanghai and Hong Kong were based on the citywide exposure metric. For the acute effects of NO_2_ on cerebrovascular and ischemic heart disease mortality, limited literatures were reported and the results are still inconsistent. According to a recent comprehensive systematic review^25^, per 10 ppb increment in NO_2_ was associated with elevated risk in stroke mortality (RR, 1.014; 95% CI: 1.009–1.019). Chen *et al*.^26^ observed a pooled estimate that a 10 μg/m^3^ increase in 2-day average concentration of NO_2_ corresponded to 1.47% (95% CI: 0.88–2.06%) increase in mortality, whereas a time stratified case crossover study of England based on MINAP database^27^ reported a 10^th^–90^th^ percentile change in 5-day moving average (lag 04) was associated with an insignificant negative effect for ischemic heart disease mortality (−0.7%; 95% CI: −3.0–1.6%). In contrast, we observed positive and significant acute effects of NO_2_ on cerebrovascular and ischemic heart disease, with the estimates of 2.07% at lag03 (95% CI: 1.23–2.91%) and 1.95% at lag02 (95% CI: 1.16–2.72%), respectively. Considering the limited number of the studies on the effects of NO_2_ on acute cardiovascular events including cerebrovascular and ischemic heart disease mortality, further studies should be conducted in elsewhere, especially in developing countries.

### Spatial variation in district-specific NO_2_ effects

For the district-specific effect estimations, only five districts were observed with positive significant association between NO_2_ and cardiovascular mortality, four districts were observed for cerebrovascular mortality and two districts for ischemic heart disease mortality. The difference in the spatial pattern of associations of NO_2_ and three interest outcomes might be partly attributed to the different aetiology and clinical characteristics of those diseases^28^. Most of the districts with significant effects were located in the centre of Beijing where tend to have a heavier traffic flow and higher population density. The strongest and significant associations between NO_2_ and three outcomes were all observed in Shijingshan and Chaoyang district. Historically, Shijingshan district was a heavily industrialized area with six coal fired power plants, iron and steel mills companies, although some of them had been moved elsewhere since 2008. Emission from coal combustion and thermal power plants was one of major sources of ambient NOx, including NO_2_, as well as particle pollutants^29^, thus the concentration of air pollutants would be higher in areas with heavy industries. Moreover, long term exposure to higher level of air pollutants could result in the injury of cardiopulmonary system via a series cascading events, such as systematic oxidative stress, dysfunction of vascular, and systematic inflammation^30^, which inevitably together increase the susceptibility of acute effects of NO_2_ for local citizens, especially for those with underlying disease (e.g. diabetes), therefore the higher estimates of NO_2_ were more likely observed in those areas. In addition, significant associations were also found in Chaoyang district. Besides the higher levels of air pollution, other factors including district-specific demographic characteristics, environmental and traffic conditions may also contribute the present observed associations in Chaoyang district. According to the Report of the Sixth National Population Census of China (2010) and the Annual Statistical Report of Beijing, the indexes of population of aged ≥65, total population, population density and total civilian vehicles were higher in Chaoyang district than the districts with lower or non-significant associations, such as Mentougou district (see Supplemental Table S1). Furthermore, the forest coverage (22.68%) of Chaoyang district was far lower than the average rate (43.49%) across Beijing during the study period. Thus the above mentioned aspects can partially explain the high estimates in Chaoyang district.

### District level indicators and spatial pattern in district-specific NO_2_ effects

We also explored whether the spatial pattern in NO_2_ related total cardiovascular, cerebrovascular and ischemic heart disease mortality risks could be explained by district level indicators. Notably, intervention targeting in high-risk districts (i.e. stronger NO_2_ effects) might varied, for instance, if the high-risk districts were those with higher proportion of illiterate population or lower educated residents versus those located in centre city with dense population and large number of vehicles. Of the 28 indicators of six dimensions, most of them showed non-significant associations with the district specific estimates of NO_2_ on cardiovascular mortality. Income inequality has been indicated as a modifier of the relationship between air pollution and health^31^. Evidence indicated that it could be a modifier of the relationship between air pollution and health and stronger air pollutants’ effects were observed in areas with lower income^32^. For present investigation, we obtained a set of indicators that could be related to district average income level, including annual GDP, per capital GDP, but we didn’t observe significant evidence that districts with higher values of these indicators had lower NO_2_ effects. The observed non-significant associations might be the results of misclassification bias because we did not obtain the detailed and accurate district-specific income data, which might hinder the investigation of effect modification by income inequality. In addition, we also didn’t find the significant association for the indicators of housing condition, a set of indicators that can also be related to district-specific income level. But the negative estimates of those indicators still indicate that there is the possibility of decreasing the district-level NO_2_ effects through the improvements in housing conditions and income level.

Coal consumption and civilian vehicles can be used as indicators of urbanization, and they have directly relationship with the increment of ambient air pollutants^33^, such as NO_2_ and PM_2.5_, and the emission control for them can partly reduce the ambient concentration of NO_2_^11^. For present investigation, we found that coal consumption and civilian vehicles could modify the district-level NO_2_ effects. It is possibly due to that the coal consumption and civilian vehicles can contribute to the differential exposures to ambient air pollutants, for instance, districts with higher value of these two indicators were tended to have higher level of particle matter and traffic related gaseous pollutants (e.g. NO_2_), resulting in higher level of NO_2_ and other pollutants among local residents. Specially, the positive correlations of district-specific average NO_2_ concentrations with coal consumption (r = 0.40, p = 0.13) and civilian vehicles (r = 0.70, p < 0.01) were observed in present study. Besides the differential exposures, another possible explanation for the effect modification by civilian vehicles was through the increased psychosocial stress among local residents. Commonly, the districts with large number of civilian vehicles were tend to have problems of dense traffic, higher traffic volume and heavier traffic noise, moreover evidences have shown that these problems triggered by heavy civilian vehicles were associated with increased perceived psychosocial stress^34^, which might further alter susceptibility to air pollution^35^. Therefore, these findings confirmed that the reduction in consumption of coal and control of vehicles may lower the risk of cardiovascular mortality among local residents.

The role of population structure and education attainment, such as total population, population density, proportion of old people and percentage of illiterate population, which constitute the important part of district- or community-specific socioeconomic status (SES)^36^ in the spatial pattern of effects of air pollutants on health outcomes has been investigated^37^,^38^, whereas no studies report results for NO_2_’s effects on cardiovascular mortality as far as we know. In our study, we found that population was positively and significantly associated with the district-specific estimates of NO_2_ on total cardiovascular and cerebrovascular mortality. Whereas positive but non-significant associations were observed for population density. Commonly, districts with large population, higher population density were likely to locate in centre of city and tended to have unfavourable built environment, which characterized by a higher amount of impervious surfaces, lower rate of green space. The unfavourable built environment might alter both the exposures and susceptibility to NO_2_ for local citizens, resulting the varied effects of NO_2_ on cardiovascular events mortalities across districts. In particular, evidences suggested the positive health impacts of higher rate of green space (e.g. forest)^39^, as well as the roles of green space in reducing level of gaseous pollutants via leaf stomata^39^. Additionally, we also observed negative but non-significantly associations for rate of forest coverage with district-level effects of NO_2_-mortality. Although we did not find significant associations with percentage of old population, we found that districts with large number of aged people were associated with higher risk of NO_2_ related cerebrovascular mortality risk. For the indicators of district-level education attainments, we observed increasing district-specific effects of NO_2_ on cerebrovascular and ischemic heart disease mortality with increasing illiterate population, but not the case for rate of illiterate population. Notably, larger illiterate population indicated the lower average education level, which was also associated with community-or district-level SES^37^. And large disparities have been observed in NO_2_, with higher exposures among residents living in lower SES areas that characterized by lower averaged education level^40^, and education level, as a part of SES, has been identified as a modifier in the associations between air pollutants and adverse health^37^,^41^. Regarding to, however, the different results for the illiterate population and proportion of this group, further studies are still needed.

A somewhat interesting finding was the observed significant negative association for marriage rate with district-level NO_2_ related cerebrovascular mortality in univariate analysis. This might be due to that the districts with lower rate of marriage more likely have higher proportion of unmarried people who are more likely suffer from higher living pressure, lack of social-emotion supporting from companion, experience of psychosocial pressure and having a higher risk of mortality^42^, ultimately resulting in higher susceptibility to NO_2_’s hazard effects than others. However, these associations were not observed in multivariable analysis. Remarkably, most of unmarried people were young, and evidence also showed that the younger age group less susceptible to effects of air pollution compared with aged people^20^. Whereas, information of combined groups of marital status and age structure in district-level was lacked in present study, which impeded the exploration of the effect modification of district-level NO_2_ effects by groups of different marital status in different age groups, resulting in, ultimately, difficulty in distinguishing the married rate from aged structure as an effect modifier for district-level NO_2_ related cardiovascular event mortalities. Therefore, further studies are still needed to strengthen the understanding of the role of district-level marital status in the spatial variation of NO_2_ effects.

According to the aforementioned findings, the identification of vulnerable districts that with higher effects of NO_2_ on cardiovascular events mortalities should depended on more comprehensive district-level indicators, which can incorporate in information of district-specific level economic status, population structure, average education level, health resources and other aspects.

There are some limitations in our study. Firstly, the daily values of NO_2_ districts without monitoring stations were estimated by established general linear models based on districts with monitoring stations. Because few meteorological factors and traffic related variables were available in our study, and the independent variable was introduced in with simple logarithmic transformation, more flexible model with more explanatory variables are needed in future studies. Secondly, we only focus on the acute effects of ambient NO_2_ and did not consider the time of individual spend indoor and outdoor, thus the district-specific concentration cannot represent the individual exposure. Thirdly, information of PM_2.5_ was not available in our study, so we cannot rule out the possibility of the confounding of PM_2.5_ since NO_2_ and PM_2.5_ share some common sources. Fourthly, when exploring the association between district-specific indicators and the spatial pattern of NO_2_ effects, we only used the annual average data of demographic and socioeconomic status from the Statistical Information Network of Beijing. Single average value of specific time period can generate information bias. Fifthly, we only investigated the associations of socioeconomic status for the NO_2_ effects in district level rather than individual level, which could introduce ecological fallacy when the results were extrapolated to individual level studies^43^. Moreover, the investigation was performed separately for each district-level indicators, comprehensive indicators that can incorporate in most information of all the separate indicators were lacked in present study, which might also hinder the generalization of our results to other study sites. Finally, because we only used two-year data, the relative short time period could lower statistical power in present investigation.

In conclusion, our results indicated that short exposure to NO_2_ was associated with elevated risks in total cardiovascular, cerebrovascular and ischemic heart disease mortality. There was spatial variation of the observed associations across the 16 districts in Beijing. We found that heterogeneity of district-specific effect estimates of NO_2_ can be partially explained by district-specific indicators. In general, districts characterized by larger population, higher consumption of coal and larger number of civilian vehicles were likely to show higher estimates of NO_2_ related total cardiovascular and cerebrovascular mortality. The effects of NO_2_ on total cardiovascular and ischemic heart disease mortality were higher in districts with larger percentage of illiterate population. The districts with more residents aged ≥65 years were also more likely to have higher risks of NO_2_ related cerebrovascular. Further investigation of district level or even precise predictors of the NO_2_ related cardiovascular mortality is warranted, which will provide insight for identifying vulnerable districts or areas and aid public health policymakers to allocate health resources efficiently.

## Methods

### Data collection

Beijing, the capital of China, is located in the northwest of Beijing-Tianjin-Hebei Delta, which is surrounded by the Yanshan Mountain in the west, north and northeast directions. The population of Beijing has raised from 17.55 million to 21.51 million from 2009 to 2014^44^. Our study period was between January 1, 2009 and December 31, 2010. And the study area included all the 16 administrative districts in Beijing, which included 6 urban districts (Dongcheng, Xicheng, Chaoyang, Fengtai, Shijingshan, Haidian), 8 suburban districts (Mentougou, Fangshan, Tongzhou, Shunyi, Changping, Daxing, Huairou, Pinggu), and 2 rural counties (Yanqing, Miyun) as depicted in Fig. 1.

Daily counts of all causes death in Beijing were obtained for the study period from the Cause of Death Registry System of Chinese Centre for Disease Control and Prevention (China CDC). The causes of death were coded according to the International Classification of Diseases, version 10 (ICD-10). Daily mortality data on deaths attributed to cardiovascular disease (I00-I99), cerebrovascular disease (I60-I69), and Ischemic heart disease (I20-I25) were used in present analyses.

Meanwhile, we also obtained the district-specific demographic and socioeconomic data from the Beijing Statistical Yearbook(2009–2010) and the 2010 Population Census of Beijing from the Beijing Statistical Information Network^44^. We obtained district-level indictors that covered 6 dimensions including economic status, population structure, education attainment of residents, health resources, housing conditions and environmental conditions (see Supplemental Table S1).

Data of daily 24-hour average of NO_2_ concentrations were collected from 12 national air quality monitoring (AQM) stations in Beijing (see Fig. 1) during the study period. To allow for the confounding effects of other pollutants on the effects of NO_2_, daily 24-hour average concentrations of PM_10_ and carbon monoxide (CO) were also collected from the 12 stations. Data from one AQM station (Dingling station in Changping district) was excluded because it was used as the background station for calibration. Specifically, the calculation of 24-hour average concentration should satisfy the criteria that at least 75% of hourly measurements per day were available and abnormal values were excluded. Citywide daily concentrations of the three pollutants were the average of the 11 stations’ concentrations. Whereas, the concentration in individual districts was based on the measured and estimated concentration. The daily concentration of pollutant of districts with more than two AQM stations was the arithmetic mean value of the 24-hours value from those stations. For districts without AQM stations, a series of general linear models (GLM) were created based on the relationships between meteorological factors, road length and measured air pollutants to estimate the relevant air pollutant daily concentration. The methodology of estimating the air pollution level in individual districts that without monitoring sites was described in detail elsewhere^5^. In brief, the estimation followed several steps: (1) dividing one year into warm period (April to September) and cold period (October to March); (2) using logarithmic CO, NO_2_ and PM_10_ concentrations as the dependent variables separately, and temperature, relative humidity, wind speed and total length of the road of each district with logarithmic transformation as independent variables to fit GLM and to estimate the regression coefficient for each pollutant in each of the two time periods; and (3) estimating the daily levels of PM_10_, NO_2_ and CO by GLM for the districts without pollution information. Because Beijing started regular fine particulate matter (PM_2.5_) monitoring only after October of 2012, no PM_2.5_ concentrations data were obtained for our study. In view of meteorological factors playing as important confounders of air pollutants-related health effects^45^,^46^, meteorological data, including daily mean temperature, relative humidity and barometric pressure in 24 h, were also obtained from the China Meteorological Administration in Beijing.

### Statistical analysis

For examining the associations between NO_2_ and citywide and district-specific cardiovascular mortalities, we used a time-stratified case-crossover design, which has become one of commonly used designs in study of acute effects of air pollution^47^,^48^,^49^. Compared with time series design, this design can control the time-invariant confounders, such as gender, smoking status, and the seasonal and secular trends by design, while the former by using smooth spline function^50^ and the results from this design for time series data were equivalent to those from the former design^51^,^52^. Additionally, the time stratified case-crossover design can be fitted by conditional logistical regression and Cox proportional hazard regression^53^, which provides computational convenience and offers an alternative design in air pollution epidemiology.

Our previous works have showed the spatial variation of air pollutants effects^5^ and other studies indicated that the health response can be clustered by location, failure to account for it can lead to an understatement of the uncertainty of the effects of air pollutants^17^. Therefore, we adopted a multilevel time stratified case crossover design with district as the random term. The estimation of the citywide effects of NO_2_ was accomplished by modelling a mixed Cox proportional hazard model. We defined the case period as the day of subject dead, and the controls days was matched on the day of the week in the same month and year as the case period. For each case day, there were 3 or 4 control days in the same month. Then we added the current day ambient temperature and humidity to control the potential confounding of these two meteorological factors. We didn’t further adjust for pressure and wind as previous studies have shown the two factors were not likely to confound the air pollutant related mortality effects^54^. Further, we constructed a set of single pollutant models with different lag structures of citywide daily mean concentrations of NO_2_, including single-day lags (from lag0 to lag3) and multi-day lags (from lag01 to lag03). For the single-day lags, lag0 corresponds to the current day concentration of NO_2_, lag1 means the concentration of the previous one day and so on. For the multi-day lags, lag01 refers to the moving average of lag0 and lag1 and so on. In order to adjust the impact of co-pollutants on the effects of NO_2_, we added the daily concentrations of PM_10_ and CO with the same lag structures to create multi-pollutants models. In order to compare the results from the traditional time stratified case-crossover design with those from multilevel time stratified case-crossover design, we also created Cox proportional hazard model without the random term of district. We also further adjusting for the temperature for 4 days (lag03) using natural cubic spline with degree of freedom of 3 to further investigate the robustness of the primary results.

For estimating district-wide effects of NO_2_, we applied the conditional logistic regression, based on the Cox proportional hazard model, with the district-level air pollution concentrations as the basic exposure metrics, and without the random effects of districts. The lag structures of NO_2_ used in district-wide model were based on their performance in the citywide analyses, i.e. the lag structures with the most significant associations between NO_2_ and the cardiovascular mortalities in the multilevel time stratified case-crossover analyses were adopted in modelling district-specific effects.

To explore the associations between spatial variation of effects of NO_2_ and the district-specific predicators, we followed several steps. Given the existence of some serious leverage and influence values among the district-specific indicators, firstly, we built robust linear regression models using the district-specific estimates of three interested mortality as the response variables and the six-dimension indicators at district level as the covariates to obtain the results, because robust regression provide resistant results in the presence of outliers^55^. Secondly, we examined each of the district-level indicator in univariate model as well as in multivariable model to identify potential predictors for the spatial heterogeneity of NO_2_’s effects. The candidate variables in the multivariable model were those who demonstrated statistically significant association in the univariate models. Finally, we used principle component analysis (PCA) to eliminate the impacts of collinearity of the combination of district level indicators when we performed the robust regression analysis. Specifically, the first two principal components that explained over 85% of total variance of the candidate variables were selected.

All the effect estimates were expressed as the percentage increase in mortality and corresponding 95% confidence intervals (CIs) per 10 μg/m^3^ increase in NO_2_ concentration. Analyses were performed using SAS (version 9.4, Cary, NC, USA) and R software (version 3.2.2) with the *survival*^56^ and *coxme*^57^ packages. Statistical significance was defined as *p* < 0.05 (two tails).

## Additional Information

**How to cite this article**: Luo, K. *et al*. Acute Effects of Nitrogen Dioxide on Cardiovascular Mortality in Beijing: An Exploration of Spatial Heterogeneity and the District-specific Predictors. *Sci. Rep.* **6**, 38328; doi: 10.1038/srep38328 (2016).

**Publisher’s note:** Springer Nature remains neutral with regard to jurisdictional claims in published maps and institutional affiliations.

## Supplementary Material

Supplementary Material

## Figures and Tables

**Table 1:** Summary statistics of daily mortality, ambient air pollutant levels and meteorological conditions in Beijing, 2009–2010*.

	Mean ± SD	Median	P_25_	P_75_	IQR
Daily mortality counts					
Cardiovascular disease	102.41 ± 20.52	100.00	87.00	117.00	30.00
Cerebrovascular disease	47.53 ± 10.31	47.00	40.00	54.00	14.00
Ischemic heart disease	46.15 ± 11.43	46.00	38.00	54.00	16.00
Pollutants					
PM_10_ (μg/m^3^)	121.04 ± 75.30	107.00	69.02	148.84	79.82
NO_2_ (μg/m^3^)	55.02 ± 24.04	50.41	39.28	64.35	25.07
CO (mg/m^3^)	1.54 ± 1.01	1.24	0.87	1.83	0.96
Meteorological factors					
Temperature(°C)	12.98 ± 11.70	14.65	1.70	24.30	22.60
Relative humidity (%)	50.98 ± 19.16	52.00	35.00	67.00	32.00
Barometric pressure(kPa)	101.20 ± 0.98	101.12	100.44	101.92	1.48

^*^SD: standard deviation; P_x_ (x^th^ percentiles), IQR: Interquartile range.

**Table 2:** The statistically significant results of univariate and multivariable analysis of estimated percent change in the association between ambient NO_2_ and mortality per unit change in the district specific indicators.

District specific indicators	Unit change in indicators	% change in risk of NO_2_-mortality (95% CI)					
		Cardiovascular		Cerebrovascular		Ischemic heart disease	
Univariate		Univariate	Multivariable*	Univariate	Multivariable*	Univariate	Multivariable*
GDP	10 billion yuan	0.05(0.00,0.10)	−0.01(−0.03,0.13)	0.12 (0.03,0.21)	0.02(0.00,0.04)#	0.02(−0.07,0.11)	—
Civilian vehicles	1 million vehicles	2.17(0.40,3.93)	0.37(0.02,0.71)	4.13 (0.86,7.97)	0.99 (0.42,1.56)	1.28(−1.66,2.13)	—
Coal consumption	1 million tons standard coal	0.21(0.08,0.34)	0.02(0.01,0.04)	0.36 (0.10,0.62)	0.06 (0.01,0.11)	0.18(−0.05,0.42)	—
Population	1 million permanent residents	0.56(0.17,0.95)	0.14(0.06,0.21)	1.23(0.50,1.95)	0.25(0.10,0.39)	0.28(−0.56,1.12)	—
Aged ≥65 years	10 thousands permanent residents	0.06(0.02,0.11)	0.01(0.00,0.02)§	0.13 (0.05,0.21)	0.03(0.01,0.04)	0.04(−0.10,0.13)	—
Illiterate population	10 thousands	1.14(0.56,1.72)	0.77(0.18,1.37)	1.41(0.18,2.64)	0.40(−0.51,1.30)	1.07(0.02,2.12)	1.07(0.02,2.12)
Married percentage	%	—	—	−0.23(−0.42,−0.03)	−0.02(−0.11,0.07)	0.10(−0.07,0.26)	—

^*^Estimates were based on multivariable robust regression that incorporate with PCA for those selected indictors.

^§^Exact estimate for indicator of aged ≥65 years and NO_2_-cardiovascular mortality in multivariable analysis was 0.009(−0.001, 0.019).

^#^Exact estimate for GDP and NO_2_-cerebrovascular mortality in multivariable analysis was 0.020(−0.004, 0.044).
