# Supplementary material for: Acute Effects of Nitrogen Dioxide on Cardiovascular Mortality in Beijing: An Exploration of Spatial Heterogeneity and the District-specific Predictors
Source: Sci Rep. 2016 Dec 2;6:38328. doi: 10.1038/srep38328 (PMC5133577; doi:10.1038/srep38328)
Supplement: Supplementary Material [file srep38328-s1.pdf]

Supplementary Material

Acute Effects of Nitrogen Dioxide on Cardiovascular Mortality in Beijing: An Exploration of Spatial Heterogeneity and the District-specific Predictors

Kai Luo[1,2], RunkuiLi[3,4], Wenjing Li[1,2], Zongshuang Wang[5], Xinming Ma[1,2], Ruiming Zhang[1,2], Xin Fang[6], Zhenglai Wu[1,2], Yang Cao[6,7]* & Qun Xu[1,2]*

[1]Department of Epidemiology and Biostatistics, Institute of Basic Medical Sciences Chinese Academy of Medical Sciences, School of Basic Medicine Peking Union Medical College, Beijing 100005, China;
[2]Centre of Environmental and Health Sciences, Chinese Academy of Medical Sciences, Peking Union Medical College, Beijing 100005, China
[3]College of Resources and Environment, University of Chinese Academy of Sciences, Beijing 100049, China;
[4]State Key Laboratory of Resources and Environmental Information System, Institute of Geographic Sciences and Natural Resources Research, Chinese Academy of Science, Beijing 100101, China;
[5]Chinese Research Academy of Environmental Sciences, Beijing 100012, China;
[6]Unit of Biostatistics, Institute of Environmental Medicine, Karolinska Institutet, Stockholm 17177, Sweden
[7]Clinical Epidemiology and Biostatistics, School of Medical Sciences, Örebro University, Örebro 70185, Sweden;
* Correspondences to:
Qun Xu, PhD
Department of Epidemiology and Biostatistics
Institute of Basic Medicine Sciences Chinese Academy of Medical Sciences & School of Basic Medicine Peking Union Medical College
Centre of Environmental and Health Sciences, Chinese Academy of Medical Sciences, Peking Union Medical College
Beijing 100005, China
Tel.: +86 10 69156403; Fax: +86 10 69156403; E-mail: xuqun@ibms.cams.cn
Yang Cao, PhD
Clinical Epidemiology and Biostatistics, School of Medical Sciences
Campus USÖ, Örebro University
Örebro 70185, Sweden
Tel: +46 19 602 6236; E-mail: yang.cao@ki.se

Table S1: Mean values (the numbers in bold represent the highest values for the specific indicators) of district-specific demographic and socioeconomic characteristics in Beijing, 2009-2010

Indicators	Unit	Districts§															
		**CP**	**CY**	**DC**	**DX**	**FS**	**FT**	**HD**	**HR**	**MTG**	**MY**	**PG**	**SJS**	**SY**	**TZ**	**XC**	**YQ**
**District Economic**																	
GDP	10 billion yuan	3.71	25.92	11.73	2.92	3.32	6.81	**26.09**	1.40	0.81	1.30	1.12	2.72	7.79	3.12	19.37	0.65
Per capita GDP	10 billion yuan/10000 permanent residents	2.88	7.70	13.14	2.31	3.57	3.46	8.19	3.71	2.83	2.82	2.67	4.45	9.66	2.73	**15.56**	2.13
Civilian vehicles	1 million vehicles	0.27	**0.77**	0.31	0.26	0.17	0.51	0.68	0.07	0.05	0.07	0.07	0.12	0.17	0.20	0.38	0.04
Per capita civilian vehicles	10 thousands vehicles/10000 permanent residents	2.02	2.28	3.42	2.04	1.85	2.61	2.14	1.86	1.88	1.40	1.54	1.90	2.09	1.74	**3.06**	1.45
Coal consumption	1 million tons standard coal	2.98	**9.40**	2.67	2.78	8.68	3.67	7.61	0.98	0.73	0.90	0.96	6.31	8.03	2.57	3.96	0.48
**Population Structure**																	
Population	1 million permanent residents	1.34	**3.36**	0.89	1.26	0.93	1.97	3.18	0.38	0.29	0.46	0.42	0.61	0.80	1.14	1.24	0.30
Population density	1000 permanent residents/km2	0.99	7.39	21.31	1.22	0.47	6.43	7.39	0.18	0.19	0.21	0.44	7.24	0.79	1.26	**24.63**	0.15
Aged ≥ 65 years	10 thousands permanent residents	9.68	**30.57**	11.32	8.23	8.31	18.69	27.51	3.26	3.20	4.79	4.37	5.95	6.94	9.14	15.76	3.15
Percent of aged ≥65 years	%	5.83	8.62	12.31	6.03	8.79	8.85	8.38	8.73	11.06	10.26	10.51	9.66	7.92	7.72	**12.68**	9.93
Sex ratio (Male vs Female)	%	115.32	106.09	97.78	**117.88**	105.79	104.27	106.83	111.95	103.57	102.29	104.87	105.47	108.02	106.42	100.29	104.28
Married percentage	%	66.52	67.55	68.52	70.17	73.29	71.98	58.55	75.74	73.79	**75.96**	74.35	70.42	72.31	71.32	66.77	69.70
**Education Attainment**																	
Percent of	%	89.78	90.86	90.08	85.22	81.06	88.37	**91.84**	78.73	80.93	76.05	75.13	88.75	84.05	85.28	89.95	74.81

High level [ǂ]																	
Percent of low level	%	10.22	9.14	9.92	14.78	18.94	11.63	8.16	21.27	19.06	23.95	24.87	11.25	15.95	14.72	10.05	**25.19**
Illiterate population[‡]	10 thousands	2.12	3.04	1.27	2.44	2.63	**2.96**	2.48	1.72	0.89	2.53	2.30	1.01	2.23	2.18	1.87	1.63
Percent of illiterate population	%	1.39	0.93	1.50	1.97	3.13	1.54	0.82	5.20	3.43	6.11	**6.20**	1.79	2.81	2.02	1.63	5.74
**Health Resources**																	
Hospital beds per 1000 permanent residents	1 bed/10 thousands permanent residents	5.35	4.39	**11.03**	2.87	5.51	3.93	2.77	3.13	8.79	1.88	3.76	4.88	2.855	1.65	10.82	2.9
Doctors per 1000 permanent residents	1 doctor/10 thousands permanent residents	2.37	3.69	**9.44**	2.23	2.52	2.42	2.78	2.92	3.55	2.64	3.02	3.43	2.58	1.98	7.85	2.66
Nurses per 1000 permanent residents	1 nurses/10 thousands permanent residents	2.31	3.78	**9.31**	1.89	2.24	2.53	2.93	2.21	3.78	1.96	2.49	3.67	2.12	1.76	9.13	1.96
Health institutes per 10 thousands permanent residents	1 institute /10 thousands permanent residents	4.35	**6.12**	5.34	3.85	5.32	2.42	2.89	4.44	4.15	4.49	2.63	3.01	3.16	2.20	4.76	2.85
Medicare expenditure rate [#]	%	4.58	6.50	**8.18**	3.06	4.93	6.52	4.77	6.26	5.05	6.39	5.48	6.95	4.77	4.31	6.59	7.05
**Housing condition**																	
Percent of households with four housing rooms	%	13.52	4.68	3.31	18.50	19.38	5.14	8.00	13.61	16.04	17.92	20.64	2.21	28.78	18.19	3.00	**40.21**
Percent of households with three housing rooms	%	35.28	23.03	19.60	34.03	43.29	22.87	30.09	43.07	33.89	42.43	61.04	19.24	48.92	35.28	21.11	**73.44**

Percent of households without kitchen	%	**18.47**	18.41	13.18	22.97	9.35	19.21	17.36	11.44	17.64	7.79	2.73	15.81	14.95	15.66	14.33	4.06
Housing rooms per household		2.58	1.99	1.87	2.90	2.78	2.04	2.17	2.45	2.64	2.52	2.81	1.96	3.41	2.73	1.87	**3.44**
**Environmental Condition**																	
Days of reach class Ⅱ air quality standard *	day	294.5	279	272.5	250	248	257.5	278.5	**310.5**	265	306	293.5	246	275	266	278.25	302.5
Percent of days of reach class Ⅱ air quality standard *	%	80.68	76.45	74.67	68.47	67.94	70.55	76.31	**85.07**	72.58	83.84	80.42	67.96	75.32	72.89	76.27	82.86
Forest coverage rate	%	60.91	22.68	19.87	25.66	53.89	38.88	42.24	**75.46**	57.09	64.44	66.25	40.12	26.56	23.37	14.51	64.06
Environment expenditure **	%	1.60	1.23	2.46	0.38	2.53	1.85	1.36	2.22	**6.06**	3.50	4.00	0.94	0.92	0.68	1.11	3.77

§CP (Changping), CY (Chaoyang), DC (Dongcheng), DX (Daxing), FS (Fangshan), FT (Fengtai), HD (Haidin), HR (Huairou), MTG (Mentougou), MY (Miyun), PG (Pinggu), SJS (Shijingshan), SY (Shunyi), TZ (Touzhou), XC (Xicheng), YQ (Yanqing) district

ł "high level" included middle schools or higher and the "low level" included primary schools or lower among the population of permanent residents aged 6 years or older.

ǂ Illiterate population meant illiterate people among the permanent residents aged 15 years or older.

# Refer to the percentage of expenditure of medicare or environmental protection in the annual financial expenditure.

* class Ⅱ air quality standard means the national ambient air quality standards of China in Class Ⅱ areas[1].

** Refer to the percentage of expenditure of environmental protection accounting for the total annual financial expenditure

Table S2. Spearman's correlation between daily ambient air Pollutants and meteorological factors

	NO_2	CO	Temperature	Relative humidity	Barometric pressure
PM_{10}	0.54	0.61	0.07	0.29	-0.22
NO_2		0.80	-0.19	0.28	0.09
CO	-		-0.24	0.47	0.08
Temperature	-	-		0.32	-0.84
Relative humidity	-	-	-		-0.31

Table S3. The Results of Univariate Analysis of Estimated Percent Change in the Association between Ambient NO_2 and Mortality per Unit Change in the District Specific Indicators#

District specific indicators	Unit change in indicators	% change in risk of NO_2-mortality (95% CI)		
		Cardiovascular	Cerebrovascular	Ischemic heart disease
**District Economic**				
GDP	10 billion yuan	0.05 (0.00, 0.10)*	0.12 (0.03, 0.21)**	0.02 (-0.07, 0.11)
Per capita GDP	10 billion yuan/10000 permanent residents	0.02 (-1.21, 1.24)	0.27 (-2.07, 2.61)	0.23 (-1.66, 2.13)
Civilian vehicles	1 million vehicles	2.17 (0.40, 3.93)*	4.13 (0.86, 7.97)*	1.28 (-2.25, 4.82)
Per capita civilian vehicles	10 thousands vehicles/10000 permanent residents	-0.13(-1.05, 0.79)	-0.20(-1.93, 1.54)	0.13(-1.35, 1.61)
Coal consumption	1 million tons standard coal	0.21(0.08, 0.34)**	0.36 (0.10, 0.62)**	0.18 (-0.05, 0.42)
**Population Structure**				
Population	1 million permanent residents	0.56(0.17, 0.95)**	1.23(0.50, 1.95)**	0.28(-0.56, 1.12)
Population density	1000 permanent residents/km^2	0.01(-0.07, 0.08)	0.02(-0.11, 0.15)	0.01(-0.10, 0.11)
Aged ≥ 65 years	10 thousands permanent residents	0.06(0.02, 0.11)**	0.13 (0.05, 0.21)**	0.04(-0.10, 0.13)
Percent of aged ≥65 years	%	-0.03(-0.29, 0.24)	-0.06(-0.56, 0.44)	0.02(-0.38, 0.43)
Sex ratio (Male vs Female)	%	-0.01(-0.11, 0.09)	0.01 (-0.17, 0.20)	-0.05(-0.19, 0.10)
Married percentage	%	-0.05(-0.16, 0.07)	-0.23(-0.42, -0.04)*	0.10(-0.07, 0.26)
**Education Attainment**				
Percent of High level ǂ	%	0.02(-0.07, 0.10)	0.07 (-0.11, 0.25)	0.02(-0.12, 0.17)
Percent of low level ǂ	%	-0.02(-0.10, 0.07)	-0.07(-0.25, 0.11)	-0.02(-0.17, 0.12)
Illiterate population ‡	10 thousands	1.12(0.56, 1.72)**	1.41 (0.18, 2.64)*	1.07(0.02, 2.12)*
Percent of illiterate population	%	-0.05 (-0.32, 0.22)	-0.19 (-0.74, 0.36)	-0.09(-0.55, 0.38)
**Health Resources**				
Hospital beds per 1000 permanent residents	1 bed/10 thousands permanent residents	-0.08(-0.24, 0.09)	-0.07(-0.40, 0.26)	-0.05(-0.31, 0.21)
Doctors per 1000 permanent residents	1 doctor/10 thousands permanent residents	-0.07(-0.31, 0.17)	-0.05(-0.51, 0.41)	-0.05(-0.42, 0.32)
Nurses per 1000 permanent	1 nurses/10 thousands	-0.04(-0.26, 0.17)	-0.02(-0.42, 0.39)	-0.03(-0.36, 0.30)

residents	permanent residents			
Health institutes per 10 thousands permanent residents	1 institute /10 thousands permanent residents	-0.11(-0.62, 0.40)	-0.15(-1.15, 0.85)	0.37 (-0.45, 1.19)
Medicare expenditure rate ǁ	%	-0.02 (-0.43, 0.38)	-0.07 (-0.81, 0.70)	0.16(-0.50, 0.81)
**Housing Condition**				
Percent of households with four housing rooms	%	-0.02(-0.07, 0.03)	-0.04(-0.14, 0.07)	-0.04(-0.11, 0.04)
Percent of households with three housing rooms	%	-0.01(-0.04, 0.03)	-0.01(-0.08, 0.06)	-0.04(-0.10, 0.02)
Percent of households without kitchen	%	-0.01 (-0.11, 0.07)	-0.02 (-0.19, 0.15)	0.05(-0.11, 0.21)
Housing rooms per household	1 housing room	-0.49 (-1.49, 0.50)	-0.89 (-3.00, 1.21)	-0.76(-2.32, 0.80)
**Environmental Condition**				
Days of reach class ǁ air quality standard §	1 day	-0.01(-0.04, 0.02)	-0.02(-0.07, 0.03)	-0.01 (-0.06, 0.03)
Percent of days of reach class ǁ air quality standard	%	-0.03(-0.13, 0.07)	-0.06 (-0.24, 0.13)	-0.05(-0.20, 0.11)
Forest coverage rate	%	-0.004 (-0.03, 0.02)	-0.01(-0.06, 0.04)	-0.01 (-0.05, 0.03)
Environment expenditure rate ǁ	%	-0.17 (-0.50, 0.16)	-0.33 (-0.98, 0.32)	-0.20 (-0.73, 0.34)

The results were from the robust regression with the MM estimation. The NO_2 exposure of the estimates for cardiovascular, cerebrovascular, ischemic heart disease mortality were based on four days moving average (lag 03), four days moving average (lag 03), three days moving average (lag 03) respectively

ł The education attainment regarded as “high level” included the middle school or higher and the “low level ” included the primary school or lower among the population of permanent residents aged 6 years or more.

ǂ The definition of illiterate population was those who were illiterate person among the population of permanent residents aged 15 years or more

ǁ Refer to the percent of expenditure of medicare or environmental protection in the annual financial expenditure.

§ class ǁ air quality standard means the national ambient air quality standards of China in Class ǁ areas[1].

* $p < 0.05$,** $p < 0.01$

Table S4. Pearson correlation coefficients for the selected district-specific indicators (coefficient/p-value)

Indicators #	Illiterate population	Population	Coal consumption	Civilian vehicle	GDP	Married percentage
Population of aged ≥65 years	0.55 (0.0283)	0.98 (<0.001)	0.63 (0.0094)	0.99 (<0.001)	0.92 (<0.001)	-0.71 (0.0019)
Illiterate population	1.00	0.61 (0.0126)	0.40 (0.1202)	0.55 (0.0286)	0.34 (0.2038)	-0.10 (0.7042)
Population		1.00	0.64 (0.0079)	0.98 (<0.001)	0.85 (<0.001)	-0.72 (0.0017)
Coal consumption			1.00	0.60 (0.0134)	0.62 (0.0109)	-0.43 (0.0995)
Civilian vehicle				1.00	0.89 (<0.001)	-0.72 (0.0018)
GDP					1.00	-0.76 (0.0006)

Indicators were statistically significant in univariate robust regression for the interest outcomes.

Table S5. Correlation matrix of the district-specific indicators involved in principle component analysis (PCA) for associations between district-specific Indicators and NO_2-cardiovascular Mortality

Indicators	Aged ≥65 years	Population	GDP	Civilian vehicles	Coal consumption	Illiterate population
Aged ≥65 years	1.000	0.976	0.918	0.992	0.627	0.547
Population	0.976	1.000	0.852	0.975	0.638	0.607
GDP	0.918	0.852	1.000	0.895	0.617	0.336
Civilian vehicles	0.992	0.975	0.895	1.000	0.603	0.546
Coal consumption	0.627	0.638	0.617	0.603	1.000	0.405
Illiterate population	0.547	0.607	0.336	0.546	0.405	1.000

Table S6. Eigenvalues of the correlation matrix of the district-specific indicators involved in principle component analysis (PCA) for associations between district-specific Indicators and NO_2-cardiovascular Mortality

Components	Eigenvalue	Difference	Proportion	Cumulative
Card1	4.613	3.887	0.769	0.769
Card2	0.724	0.193	0.121	0.889
Card3	0.532	0.424	0.089	0.978
Card4	0.107	0.088	0.018	0.996
Card5	0.019	0.013	0.003	0.999
Card6	0.006		0.001	1.000

Table S7. Eigenvectors of the six components (card1-card6) involved in principle component analysis (PCA) for associations between district-specific Indicators and NO_2-cardiovascular Mortality

Indicators	Card1	Card2	Card3	Card4	Card5	Card6
Aged ≥65 years	0.458	-0.104	-0.193	-0.112	0.257	-0.815
Population	0.455	0.012	-0.156	-0.464	-0.733	0.123
GDP	0.421	-0.391	-0.103	0.776	-0.190	0.144
Civilian vehicles	0.454	-0.091	-0.230	-0.278	0.597	0.547
Coal consumption	0.340	-0.048	0.935	-0.072	0.057	0.004
Illiterate population	0.292	0.909	-0.038	0.295	0.020	0.022

Table S8. Correlation matrix of the district-specific indicators involved in principle component analysis for associations between district-specific Indicators and NO_2-cerebrovascular Mortality

Indicators	Aged ≥65 years	Population	GDP	Civilian vehicles	Coal consumption	Illiterate population	Marriage percentage
Aged ≥65 years	1.000	0.976	0.918	0.994	0.627	0.547	-0.714
Population	0.976	1.000	0.852	0.975	0.638	0.607	-0.720
GDP	0.918	0.852	1.000	0.895	0.617	0.336	-0.764
Civilian vehicles	0.992	0.975	0.895	1.000	0.603	0.546	-0.717
Coal consumption	0.627	0.638	0.617	0.603	1.000	0.404	-0.427
Illiterate population	0.547	0.607	0.336	0.546	0.404	1.000	-0.103
Marriage percentage	-0.714	-0.720	-0.764	-0.717	-0.427	-0.103	1.000

Table S9. Eigenvalues of the correlation matrix of the district-specific indicators involved in principle component analysis (PCA) for associations between district-specific Indicators and NO_2-cerebrovascular Mortality

	Eigenvalue	Difference	Proportion	Cumulative
Cere1	5.142	4.179	0.735	0.735
Cere2	0.963	0.418	0.137	0.872
Cere3	0.544	0.318	0.078	0.950
Cere4	0.226	0.121	0.032	0.982
Cere5	0.105	0.090	0.015	0.997
Cere6	0.015	0.010	0.002	0.999
Cere7	0.005		0.001	1.000

Table S10. Eigenvectors of the seven components (Cere1-Cere7) involved in principle component analysis (PCA) for associations between district-specific Indicators and NO_2-cerebrovascular Mortality

	Cere1	Cere2	Cere3	Cere4	Cere5	Cere6	Cere7
Aged ≥65 years	0.434	0.002	-0.136	0.272	0.169	-0.102	-0.826
Population	0.431	0.067	-0.142	-0.015	0.445	0.730	0.241
GDP	0.407	-0.231	0.016	0.413	-0.738	0.183	0.180
Civilian vehicles	0.430	0.001	-0.178	0.232	0.320	-0.635	0.472
Coal consumption	0.314	0.150	0.926	-0.117	0.066	-0.060	-0.006
Illiterate population	0.249	0.794	-0.240	-0.363	-0.337	-0.068	-0.005
Marriage percentage	-0.338	0.537	0.121	0.746	0.106	0.106	0.057

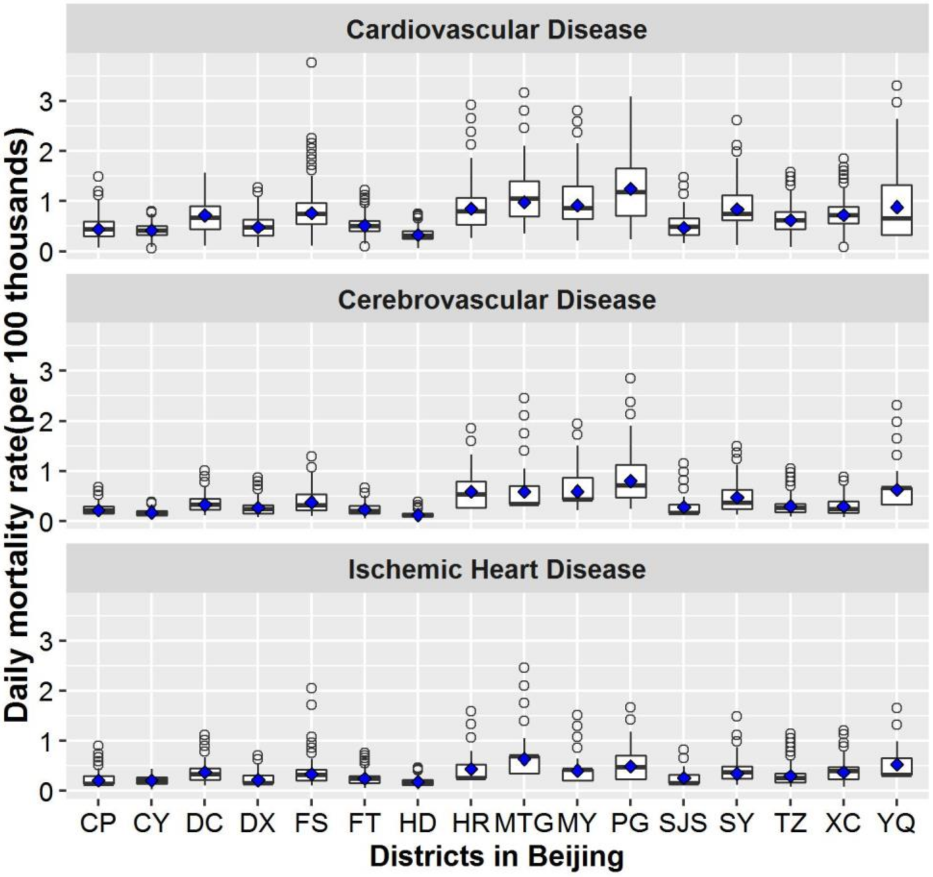

Figure S1. Box plots of district specific daily mortality rate of cardiovascular, cerebrovascular, ischemic heart disease in Beijing, 2009-2010. Boxes, lines within the boxes, blue diamonds and the circle dots indicate the interquartile range (25% to 75%), median, mean, and outliers, respectively. The abbreviations that CP, CY, DC, DX, FS, FT, HD, HR, MTG, MY, PG, SJS, SY, TZ, XC, YQ represent Chaoyang, Changping, Dongcheng, Daxing, Fangshan, Fengtai, Haidin, Huairou, Mentougou, Miyun, Pinggu, Shijingshan, Shunyi, Tongzhou, Xicheng, Yanqing district in Beijing, respectively.

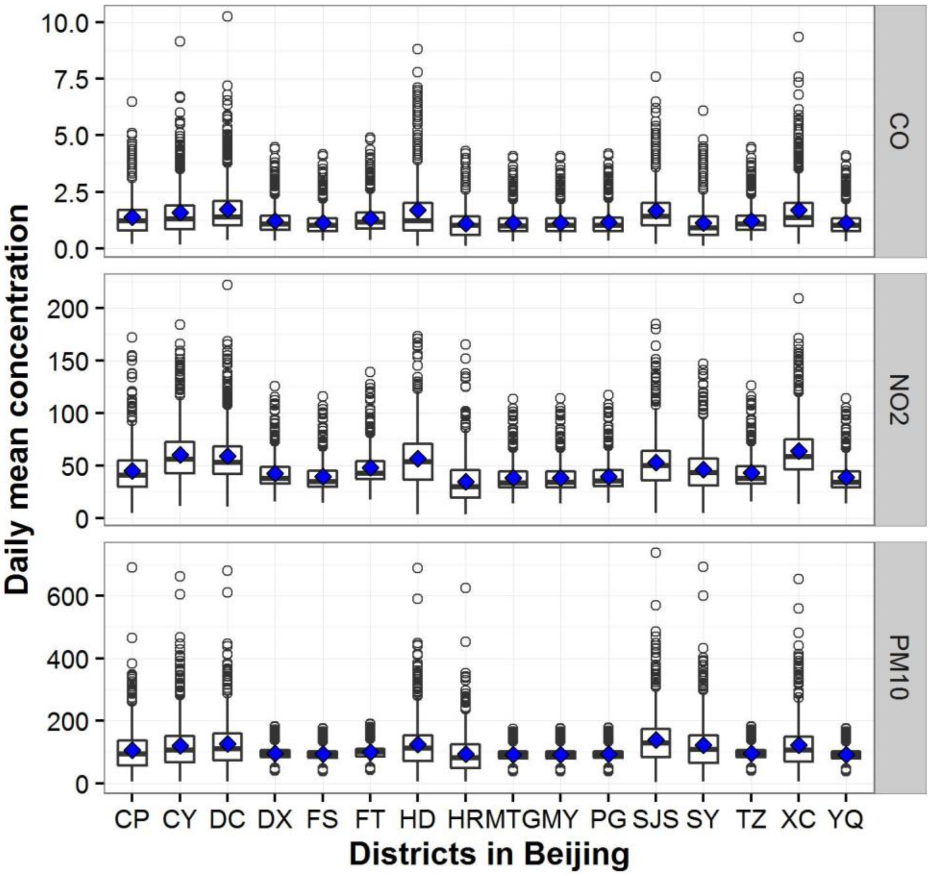

Figure S2: Box plots of district specific daily mean concentration of NO_2, PM_{10} (μg/m^3) and CO (mg/m^3), 2009-2010. Boxes, lines within the boxes, blue diamonds and the circle dots indicate the interquartile range (25% to 75%), median, mean, and outliers, respectively. The abbreviations that CP, CY, DC, DX, FS, FT, HD, HR, MTG, MY, PG, SJS, SY, TZ, XC, YQ represent Chaoyang, Changping, Dongcheng, Daxing, Fangshan, Fengtai, Haidin, Huairou, Mentougou, Miyun,Pinggu, Shijingshan, Shunyi, Tongzhou, Xicheng, Yanqing district in Beijing ,respectively.

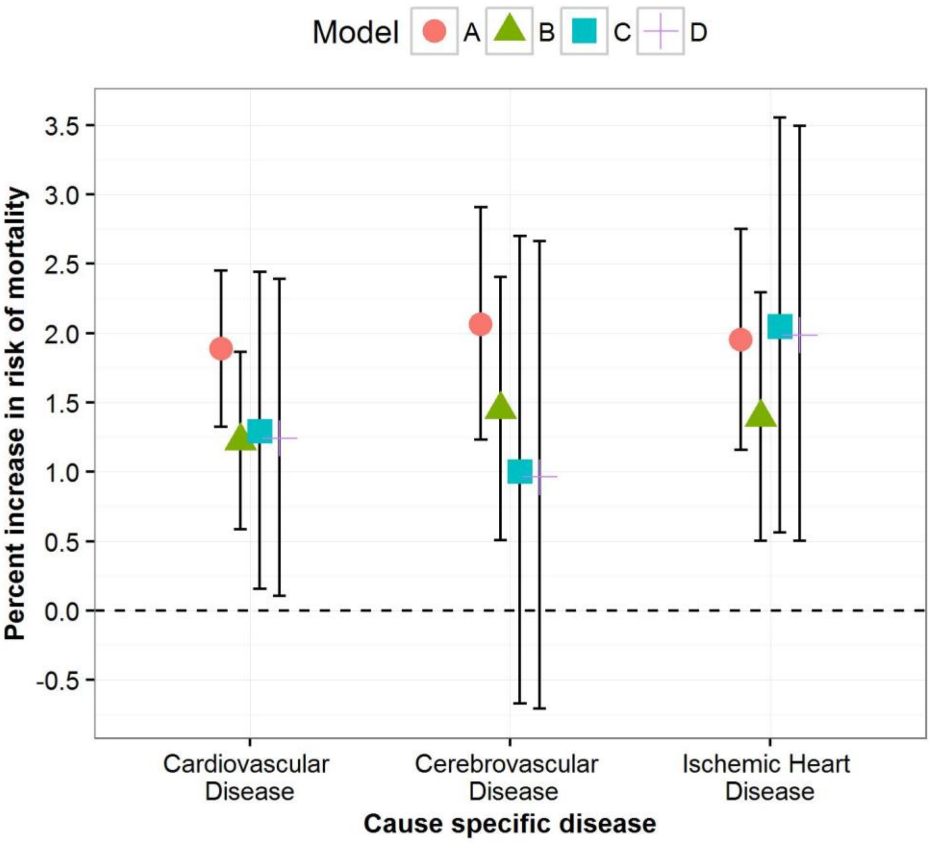

Figure S3: Citywide association between NO_2 and mortality of death from total cardiovascular, cerebrovascular, and ischemic heart disease based on single pollutant models and multi-pollutant models from multilevel time stratified case crossover analyses. The abbreviations of A, B, C, D represent single pollutant (only for NO_2) model, model adjusted for PM_{10}, model adjusted for CO, model adjusted for PM_{10} and CO. Effect estimations were based on 10 μg/m^3 increase in NO_2 for cardiovascular(lag 03),cerebrovascular(lag 03) and ischemic heart disease(lag 02)

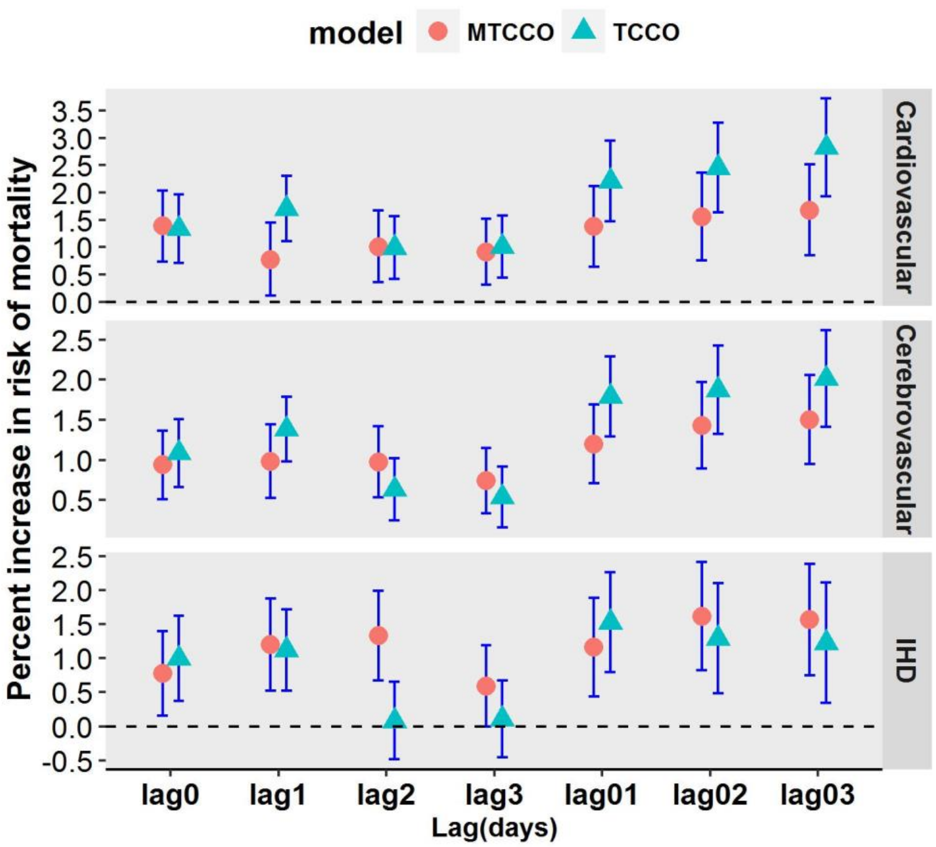

Figure S4: Estimated effects of NO_2 from MTCCO and TCCO after further adjusting for temperature for four days (lag03).

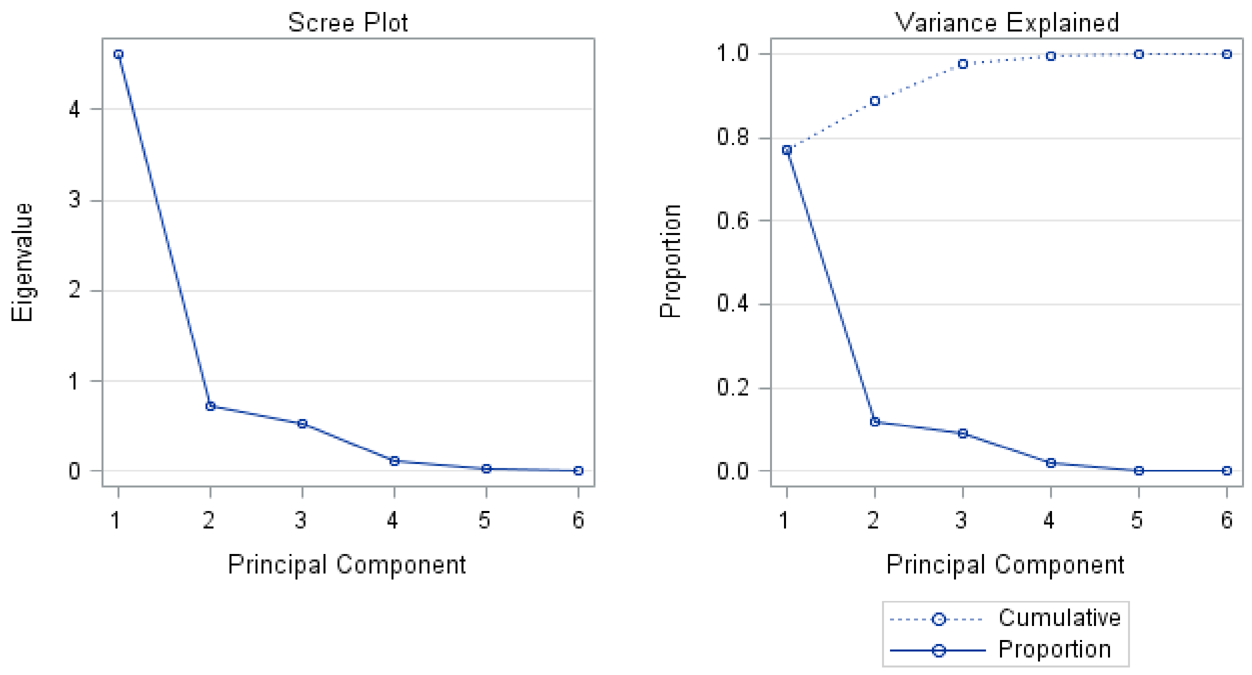

Figure S5: Scree plot for the six components for the selected indicators in principle components analysis (PCA) for associations between district-specific indicators and NO_2-cardiovascular mortality

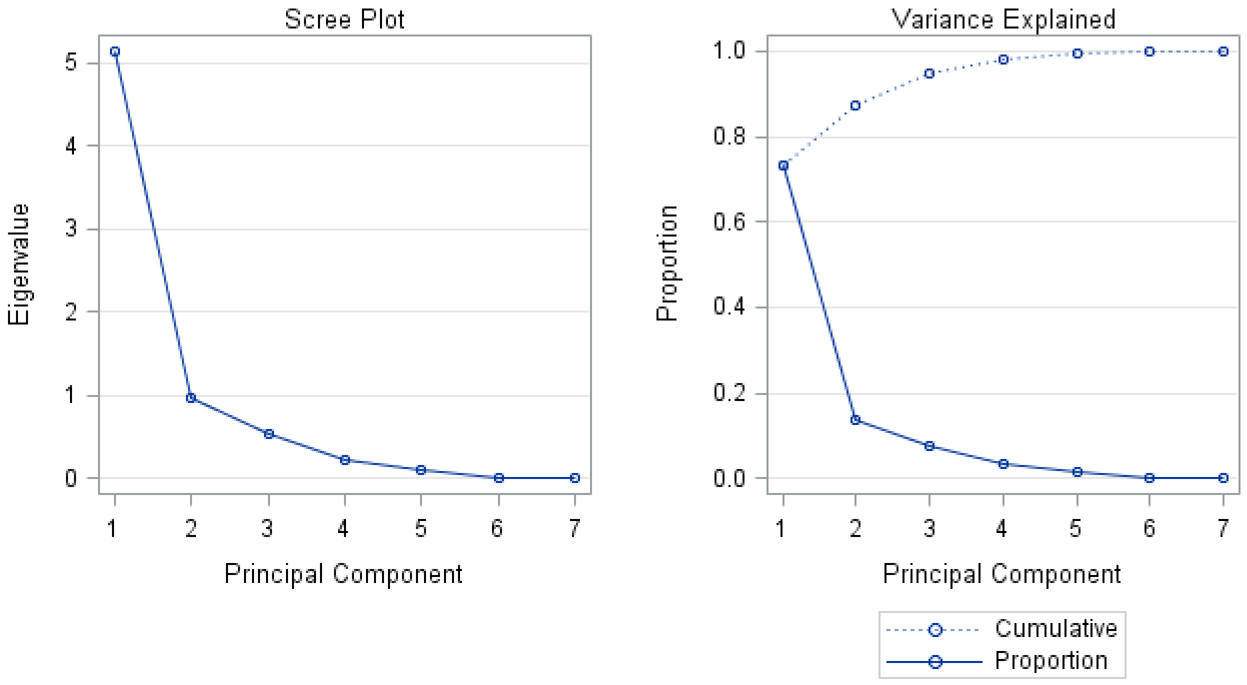

Figure S6: Scree plot for the six components for the selected indicators in principle components analysis for associations between district-specific indicators and NO_2-cerebrovascular mortality

References

1 *China National Ambient Air Quality Standard*, (1996). Available at : http://www.mep.gov.cn/image20010518/5298.pdf. (Acessed:11 th November 2015).
